# Optimization of 2D Ag-MBene-Modified Polyethersulfone Ultrafiltration Membranes for Enhanced Water Treatment Performance

**DOI:** 10.3390/membranes16090303

**Published:** 2026-09-15

**Authors:** Asam Amin Almulla, Fikri T. Dweiri

**Affiliations:** Department of Industrial Engineering and Engineering Management, University of Sharjah, Sharjah 27272, United Arab Emirates

**Keywords:** Ag-MBene, polyethersulfone, ultrafiltration, antifouling, wastewater treatment

## Abstract

Modified PES ultrafiltration membranes containing Ag-MBene nanoparticles were developed via the non-solvent-induced phase separation (NIPS) technique and evaluated for tertiary-treated sewage (TSE) filtration applications. This study aims to establish an integrated understanding of membrane structure, properties, and performance by correlating morphology, roughness, hydrophilicity, porosity, permeability, antifouling properties, and pollutant rejection capabilities. Ag-MBene nanomaterials were incorporated into the PES membrane at various loading concentrations to increase the membrane hydrophilicity and antifouling characteristics. The detailed characterization of membrane samples was performed using SEM, AFM, EDS, FTIR, contact angle analysis, and porosity evaluation. The membranes exhibited distinct structural features after the addition of Ag-MBene nanomaterials. SEM analysis revealed enhanced macropore formation and increased pore interconnectivity at a relatively moderate loading concentration of nanoparticles. AFM analysis further confirmed progressive membrane roughness enhancement after incorporating Ag-MBene particles. The contact angle decreased from 81.2° (UF00) to 47.3° (UF1.5), indicating enhanced hydrophilicity of the modified membranes. UF0.5 exhibited the most balanced overall performance due to optimized pore morphology, improved wettability, and uniform nanoparticle dispersion. The modified membranes also exhibited enhanced flux performance, more reversible fouling behavior, and improved chemical oxygen demand (COD)/total suspended solids (TSS) removal efficiency than pristine membranes. ANOVA revealed significant differences among various membranes (*p* < 0.05), and the UF0.5 configuration was identified as the optimal membrane based on its balanced structural and performance characteristics. These findings demonstrate that balanced optimization of membrane morphology, hydrophilicity, and nanoparticle dispersion is essential for maximizing ultrafiltration performance.

## 1. Introduction

Global freshwater shortages are becoming increasingly severe as a result of rapid industrial growth, urban expansion, rising population levels, and climate change [1]. The growing stress on traditional freshwater resources has encouraged the advancement of sustainable wastewater treatment and water reuse technologies that can meet long-term environmental and industrial requirements. Wastewater reclamation and reuse are now widely recognized as effective approaches for alleviating pressure on freshwater reserves while promoting sustainable water resource management practices.

Among the various advanced treatment methods, membrane-based separation technologies have gained significant interest because of their compact structure, high separation capability, ease of operation, scalability, and lower chemical consumption [2]. Membrane systems are extensively applied in desalination, wastewater treatment, industrial water purification, and tertiary polishing processes, attributable to their efficiency in removing suspended solids, colloidal materials, microorganisms, macromolecules, and organic pollutants [3].

Ultrafiltration (UF) membranes are especially promising for tertiary wastewater treatment since they function under relatively low hydraulic pressure while still achieving high contaminant removal performance [4]. UF membranes can effectively eliminate turbidity, suspended solids, organic contaminants, bacteria, and colloidal substances from wastewater streams, making them highly appropriate for water reuse purposes [2,5]. Furthermore, UF systems exhibit stable operational behavior and can be combined with advanced oxidation, adsorption, or reverse osmosis processes in integrated multi-stage treatment systems.

Despite these benefits, membrane fouling continues to be one of the primary challenges limiting the long-term efficiency of ultrafiltration systems because it causes flux decline, pore blockage, and increased hydraulic resistance [6]. Fouling causes substantial permeability loss, pore obstruction, increased transmembrane pressure, greater energy demand, and reduced membrane service life [7]. During filtration, organic foulants, suspended particles, colloidal substances, and microbial species accumulate on membrane surfaces and within membrane pores, leading to flux reduction and irreversible structural damage. Therefore, the development of antifouling ultrafiltration membranes with improved hydrophilicity and optimized pore structures has become a major focus in membrane research.

Polyethersulfone (PES) membranes are widely recognized as some of the most frequently utilized polymeric materials for ultrafiltration applications due to their outstanding thermal stability, strong mechanical properties, chemical durability, and ease of fabrication [4]. These membranes can be conveniently prepared through phase inversion techniques and maintain stable structural integrity under various operating environments. Owing to these characteristics, PES membranes are extensively applied in wastewater treatment and industrial separation processes.

Despite these advantages, unmodified PES membranes inherently exhibit hydrophobic properties that contribute to foulant attachment and membrane fouling [7]. Hydrophobic membrane surfaces tend to facilitate the adsorption of organic substances and microbial contaminants, causing significant permeability loss and rapid flux deterioration during filtration operations [6,7]. Furthermore, membrane fouling increases hydraulic resistance, lowers filtration performance, and necessitates frequent cleaning or membrane replacement.

The fouling behavior of membranes is highly dependent on several factors, including surface chemistry, surface roughness, hydrophilicity, pore size distribution, porosity, and internal structural characteristics [7]. Membranes with dense surfaces and poor pore interconnectivity may hinder water transport and promote contaminant accumulation within the membrane matrix. Likewise, excessive surface roughness can create depressions and protruding regions that retain foulants and intensify irreversible fouling. Therefore, precise control of membrane morphology and surface characteristics is crucial for enhancing permeability and minimizing fouling tendencies.

Numerous membrane modification techniques have been explored to enhance the hydrophilicity and antifouling properties of PES ultrafiltration membranes [8,9,10]. These strategies include surface coating, graft polymerization, blending with hydrophilic additives, and embedding nanomaterials into the polymer matrix. Among these methods, the incorporation of nanomaterials has become one of the most promising approaches because it can simultaneously improve membrane morphology, pore structure, hydrophilicity, permeability, and resistance to fouling [6,7].

The integration of nanomaterials into polymeric membrane matrices has attracted substantial interest in recent years because of their capability to enhance membrane physicochemical characteristics and separation efficiency [11]. Nanoparticles can influence membrane surface chemistry, modify phase inversion behavior, improve pore formation, and increase membrane wettability. Together, these effects contribute to enhanced permeability and improved resistance to membrane fouling.

A wide range of nanomaterials, such as graphene oxide, MXenes, metal oxides, carbon nanotubes, transition metal carbides, and other two-dimensional layered materials, have been explored for membrane modification purposes [12]. Among them, two-dimensional nanostructured materials have demonstrated remarkable potential because of their large surface area, layered architecture, adjustable surface chemistry, and abundance of functional groups.

MBenes are an emerging class of two-dimensional transition metal borides distinguished by their layered structures, high surface reactivity, and excellent hydrophilic nature [13]. Their distinctive morphology and chemical properties make them highly promising for membrane modification applications. Incorporating MBene into polymeric membranes can improve membrane hydrophilicity, enhance pore interconnectivity, decrease hydraulic resistance, and mitigate fouling behavior [14,15].

Recent studies on nanocomposite PES ultrafiltration membranes incorporating graphene oxide, MXenes, silver nanoparticles, and other two-dimensional materials have demonstrated significant improvements in permeability, hydrophilicity, and fouling resistance. Nevertheless, the use of Ag-MBene nanostructures in PES ultrafiltration membranes remains largely unexplored, particularly for TSE applications [14,15,16,17].

Beyond the inherent advantages of MBene, the addition of silver nanoparticles can further enhance membrane functionality. Chen et al. reported that silver-modified nanomaterials exhibit potential antibacterial activity, as reported in previous studies, and are capable of minimizing biofouling during wastewater treatment operations [16]. Moreover, Ag-MBene nanoparticles can improve membrane wettability and facilitate water transport by strengthening hydrophilic interactions between the membrane surface and water molecules.

Previous research has extensively explored nanomaterial-enhanced ultrafiltration membranes for water treatment applications. Studies by Rong et al. and Liu et al. reported notable enhancements in membrane permeability, antifouling properties, contaminant removal efficiency, and hydrophilicity after incorporating nanomaterials into the membrane matrix [17,18]. However, improvements in membrane performance do not necessarily increase proportionally with higher nanoparticle concentrations. An excessive amount of nanoparticles can elevate the viscosity of the casting solution, restrict solvent-non-solvent exchange, and encourage nanoparticle agglomeration. These effects may lead to the formation of dense membrane structures, partial pore occlusion or collapse, and ultimately lower membrane permeability [16,19]. Consequently, identifying the optimal nanoparticle loading is crucial to achieving a balance between permeability, hydrophilicity, and membrane structural integrity [17].

Although numerous studies have focused on nanomaterial-modified ultrafiltration membranes, only a limited number have systematically investigated the relationship between membrane morphology, surface roughness, hydrophilicity, porosity, permeability, fouling characteristics, and pollutant rejection in tertiary wastewater treatment applications [19]. Moreover, the integration of statistical validation techniques with comprehensive membrane performance evaluation for membrane optimization has received relatively limited attention in the literature [20]. Additionally, the use of Ag-MBene nanoparticles to modify PES ultrafiltration membranes for tertiary wastewater treatment has not yet been thoroughly examined.

Unlike previous studies that primarily focused on permeability enhancement or contaminant removal, the present work establishes an integrated structure-property-performance framework by correlating membrane morphology, roughness, hydrophilicity, porosity, pore size, permeability, fouling resistance, and pollutant rejection. Furthermore, the incorporation of ANOVA provides a systematic approach for statistically validating membrane performance and identifying the optimal nanoparticle loading concentration.

The novelty of the present study further lies in combining membrane characterization, filtration performance evaluation, and ANOVA statistical validation within a unified framework. This approach enables identification of the optimal nanoparticle loading while simultaneously establishing mechanistic relationships between membrane structure, surface properties, and filtration performance.

## 2. Materials and Methods

### 2.1. Chemicals and Materials

Polyethersulfone (PES, average molecular weight = 58,000 g mol^−1^, Sigma-Aldrich, St. Louis, MO, USA) pellets were used as the membrane-forming polymer. Polyvinylpyrrolidone (PVP, Mw = 40,000 g mol^−1^, Sigma-Aldrich, USA) was used as the pore-forming additive, while *N*-Methyl-2-pyrrolidone (NMP, 99% purity, Sigma-Aldrich, USA) was used as the solvent. Ag-MBene nanoparticles were synthesized according to the procedure described in our previous work and incorporated into the PES matrix at different loading concentrations.

The MBene nanostructures were synthesized from MoAlB precursor through selective etching and exfoliation processes. Silver nanoparticles were subsequently immobilized onto the MBene surface through a reduction-assisted functionalization route. The resulting Ag-MBene powder was washed, dried, and stored under ambient conditions before membrane fabrication. Detailed synthesis procedures and structural characterization are available in our previous work.

Ag-MBene refers to silver-functionalized two-dimensional MBene nanosheets, where silver species are immobilized onto the MBene surface to enhance hydrophilicity and potentially improve biofouling resistance. The modification was achieved through physical incorporation into the PES casting solution during membrane fabrication.

All chemicals and reagents used in this study were used without additional purification and were of analytical grade purity. DI water was used throughout membrane fabrication and filtration experiments. TSE was used as the feed solution for membrane fouling studies and filtration tests.

The TSE samples were collected from the tertiary treatment stage of the Sharjah Municipality Wastewater Treatment Plant, Sharjah, UAE. The treatment train consisted of primary sedimentation, biological secondary treatment, secondary clarification, sand filtration, and chlorination prior to sample collection. The collected TSE was stored at 4 °C and used within 24 h of collection.

The Ag-MBene nanomaterial employed in this study was synthesized and comprehensively characterized in our previous work, including XRD, SEM, TEM, XPS, particle-size distribution, and surface chemistry analyses. Since the objective of the present study is membrane fabrication and performance evaluation rather than nanomaterial synthesis, only a concise description is presented here.

### 2.2. Material Preparation

Fabrication of Ag-MBene PES ultrafiltration membranes was performed via the NIPS technique. Initially, a PES polymer was dissolved in NMP solvent in the presence of continuous stirring to obtain a homogeneous polymer solution. g–MBene nanoparticles were incorporated into the casting solutions at concentrations of 0.1, 0.3, 0.5, 1.0, and 1.5 wt.% of the total casting formulation. The PES and PVP contents were maintained at 16 and 2 wt.%, respectively, while the NMP content was adjusted accordingly to maintain a total casting-solution composition of 100 wt.%.

After nanoparticle addition, the casting solution was continuously stirred at 60 °C for 24 h and subsequently ultrasonicated for 30 min to promote uniform nanoparticle dispersion and minimize agglomeration. Prior to the membrane casting, all solutions were degassed to avoid the entrapment of air bubbles in the solution.

The polymer solution was subsequently cast on a clean glass plate using the casting knife with a wet casting thickness of 200 μm. Immediately after casting, the membrane film was immersed in the DI water bath as the non-solvent to perform the phase inversion procedure. In this procedure, solvent-non-solvent exchange caused polymer phase separation and membrane formation. Finally, fabricated membranes were extensively washed using DI water to eliminate solvent and other non-reacted species, and were stored in DI water for characterization and filtration studies.

Table 1 summarizes the composition of the casting solutions used for membrane fabrication. The concentrations of PES and PVP were maintained constant at 16 wt.% and 2 wt.%, respectively, while the Ag-MBene loading was varied from 0 to 1.5 wt.% by adjusting the NMP content accordingly. This approach enabled systematic investigation of the effect of Ag-MBene incorporation on membrane morphology, hydrophilicity, porosity, and antifouling performance.

### 2.3. Membrane Characterization

#### 2.3.1. Surface and Cross-Sectional Morphology

Membrane surface and cross-sectional morphologies were investigated by means of field emission scanning electron microscopy (FE-SEM). Prior to the imaging process, membrane samples were coated with a thin conductive gold layer.

Membrane cross-sections were prepared by immersion in liquid nitrogen followed by fracture. Membrane pore morphology, macro void formation, interconnectivity of pores, structural homogeneity, and the effect of Ag-MBene incorporation on membrane structure evolution were evaluated using FE-SEM analysis.

#### 2.3.2. Energy-Dispersive Spectroscopy (EDS)

Energy-dispersive spectroscopy (EDS) analysis was performed to evaluate elemental composition and nanoparticle distribution within the membrane matrix. It was used to evaluate the presence and spatial distribution of Ag after its incorporation into the polyethersulfone (PES) membrane structure. Elemental mapping was also carried out to observe how evenly the nanoparticles were distributed across the membrane surface. This helped assess whether the nanoparticles were well-dispersed or tended to cluster during membrane formation.

#### 2.3.3. Atomic Force Microscopy (AFM)

Atomic force microscopy (AFM) analysis was conducted for the study of the surface topography and roughness characteristics of membrane samples incorporated with Ag-MBene nanoparticles. Surface roughness was evaluated using the average roughness parameter (Ra), which was selected as the primary indicator for comparing membrane topography and correlating roughness with filtration performance.

The AFM analysis of surface roughness evolution was conducted using a scanning area of 5 µm × 5 µm.

#### 2.3.4. Fourier Transform Infrared Spectroscopy (FTIR)

Fourier transform infrared spectroscopy (FTIR) was applied to examine the presence of functional groups within the membranes and to investigate the interaction of the PES matrix with Ag-MBene nanoparticles.

#### 2.3.5. Contact Angle Analysis

The fabricated membranes’ hydrophilicity was tested by measuring the water contact angles statically. A water droplet was deposited onto the membrane, and the angle was taken right upon contact.

Measurements were repeated at multiple membrane locations and averages were taken. A lower value of contact angle reflects an increase in hydrophilicity and interaction between the membranes and water.

#### 2.3.6. Porosity Measurement

Membrane porosity was determined by the gravimetric wet-dry technique. Initially, membrane samples were completely wetted using distilled water. After removing excess water from the membranes’ surface, their wet weight was measured.

Afterwards, they were dried inside the oven until their dry weight was stabilized. Membrane porosity was calculated using Equation (1):

ε = ((Ww − Wd)/(ρwAl)) × 100(1)

where ε is membrane porosity (%), Ww and Wd are wet and dry membrane weights, respectively, ρw is water density, A is membrane area, and l is membrane thickness. The porosity was determined using the gravimetric method reported previously.

The average thickness of the fabricated membranes was approximately 200 μm.

#### 2.3.7. Mean Pore Size Determination

##### Mean

The mean pore radius of the fabricated membranes was estimated using the Guerout–Elford–Ferry equation:

(2) $$rm\;=\;\sqrt{[}\left(2.9-1.75\epsilon \right)8\eta lQ/\varepsilon A\Delta P]$$

where r is pore radius, ε is membrane porosity, η is water viscosity, l is membrane thickness, Q is permeate flow rate, A is membrane area, and ΔP is transmembrane pressure.

Pore size of the membrane was estimated using the Guerout–Elford–Ferry equation depending on the porosity and pure water flux. This method provides an indirect but well-established way to approximate pore size in ultrafiltration membranes.

It is particularly useful for linking transport performance with the underlying membrane structure in a practical and consistent manner.

### 2.4. Membrane Filtration Performance

Membrane performance was assessed based on a cross-flow ultrafiltration system run in a controlled hydraulic regime. Prior to wastewater filtration experiments, membranes were compacted with DI water at an operating pressure of 1 bar for approximately 30 min until a stable permeate flux was achieved. Compaction minimized membrane structural relaxation and ensured reproducible permeability measurements. All flux measurements were performed under identical operating pressure and crossflow conditions to ensure comparability among membrane configurations. Filtration experiments were conducted using a Sterlitech CF042SS crossflow filtration unit with an effective membrane area of 0.0042 m^2^. The membranes were compacted using DI water prior to testing until stable flux conditions were achieved. Experiments were performed at a transmembrane pressure of 1 bar, a feed flow rate of 300 mL min^−1^, and a temperature of 25 ± 1 °C. The feed circulation was maintained using a Kamoer KK1800 peristaltic pump operating at approximately 128 rpm.

There were three main steps in the filtration tests:

1. Permeate flux test using pure water;
2. TSE fouling step;
3. DI water recovery step.

Firstly, the pure water flux test was carried out to estimate the intrinsic permeability of the membranes before fouling. Next, TSE was applied to the filtration system to assess fouling tendency, flux reduction rate, and pollutant rejection efficiency.

Finally, after fouling, the membranes were cleaned using DI water and then filtered again to assess both reversible and irreversible fouling behavior.

Membrane flux was determined by the formula below:

(3) $$J=\frac{V}{A\times t}$$

where J is permeate flux (LMH), V is permeate volume (L), A is membrane area (m^2^), and t is filtration time (h). Flux calculations were performed according to conventional ultrafiltration methodology [5].

### 2.5. Fouling Analysis

The fouling characteristics of the membranes were investigated based on their flux recovery ratio (FRR), reversible fouling ratio (Rr), irreversible fouling ratio (Rir), and total fouling ratio (Rt).

(4) $$FRR=\frac{J_{w2}}{J_{w1}}\times 100$$

(5) $$R_{r}=\frac{J_{w2}-J_{p}}{J_{w1}}\times 100$$

(6) $$R_{ir}=\frac{J_{w1}-J_{w2}}{J_{w1}}\times 100$$

(7) $$R_{t}=\frac{J_{w1}-J_{p}}{J_{w1}}\times 100$$

where Jw1 is the initial pure water flux, Jp is the fouled flux, and Jw2 is the recovered water flux. Equations (4)–(7) were employed to evaluate flux recovery ratio, reversible fouling, irreversible fouling, and total fouling according to previous studies [10].

Flux recovery ratio was considered to evaluate how efficient the cleaning process is for antifouling behavior assessment and improvement of the membrane cleaning performance.

The reversible fouling ratio evaluates the ability of weakly bound fouling contaminants to be detached from the membrane by washing, whereas irreversible fouling refers to strongly attached foulants leading to an irreversible permeability loss.

The total fouling ratio is the sum of all contaminant deposits on the membrane during filtration.

### 2.6. COD and TSS Removal Performance

Membrane pollutant rejection efficiency during tertiary wastewater treatment was investigated via COD and TSS analyses of tertiary wastewater filtration. Samples of feed and permeate were taken at different times and analyzed to assess the organic pollutant and suspended solid rejection efficiencies of the membranes.

Organic pollutant rejection efficiency was measured as a COD rejection efficiency, whereas suspended solid rejection efficiency was determined as TSS rejection efficiency.

Chemical oxygen demand (COD) was determined according to Standard Methods for the Examination of Water and Wastewater (APHA), whereas total suspended solids (TSS) were measured gravimetrically using pre-weighed filter papers followed by drying at 105 °C to constant weight.

Rejection efficiency is determined according to the following formula:

(8) $$R\;(\%)\;=\;\frac{C_{f}-C_{p}}{C_{f}}\;\times \;100$$

where Cf and Cp represent feed and permeate concentrations, respectively. Rejection efficiency was calculated according to conventional membrane separation studies [5].

### 2.7. Statistical Analysis

Statistical tests were carried out to examine differences in membrane performance among the different membranes prepared. The test carried out was one-way analysis of variance (ANOVA) for the determination of statistical significance on permeability, fouling resistance, recovery properties, and pollutant rejection.

The significance value was set at *p* < 0.05. Statistical significance was determined in order to show that differences in performance observed resulting from the inclusion of Ag-MBene in membranes were statistically significant.

All experiments were conducted in triplicate, and results are reported as mean ± standard deviation. As shown in Table 2, the one-way ANOVA confirmed statistically significant differences among membrane configurations. Although post hoc multiple-comparison tests such as Tukey’s Honest Significant Difference (HSD) analysis were not performed in the present work, future investigations may employ such analyses to further distinguish statistically significant differences between individual membrane pairs.

One-way ANOVA confirmed statistically significant overall differences among membrane configurations for average flux, flux recovery ratio (FRR), COD removal, and TSS rejection (*p* < 0.05). UF0.5 exhibited the highest overall experimental performance among the investigated membrane configurations. However, because post hoc multiple-comparison testing was not performed, the ANOVA results are interpreted as evidence of overall differences among membrane configurations rather than statistical significance between specific membrane pairs.

### 2.8. Integrated Membrane Performance Evaluation

Due to the inherently conflicting nature of membrane performance parameters, where permeability, fouling resistance, and solute rejection exhibit trade-off relationships, single-parameter optimization is insufficient for identifying the optimal membrane configuration. Therefore, an integrated performance evaluation approach was used to consider all key performance indicators within a unified assessment framework.

Because membrane performance involves multiple interconnected criteria, including permeability, fouling resistance, contaminant rejection, and membrane stability, the experimental data were organized and compared across the selected membrane configurations. The evaluation considered the relative performance of each membrane based on normalized trends in the measured indicators, allowing differences among performance parameters to be assessed consistently. Membranes showing balanced improvement across permeability, rejection efficiency, fouling resistance, recovery behavior, and structural stability were considered to possess superior overall performance. This approach enables simultaneous evaluation of multiple performance parameters while identifying the most balanced membrane configuration.

## 3. Results and Discussion

### 3.1. SEM Analysis

#### 3.1.1. Surface Morphology Evolution

**Figure 1:**
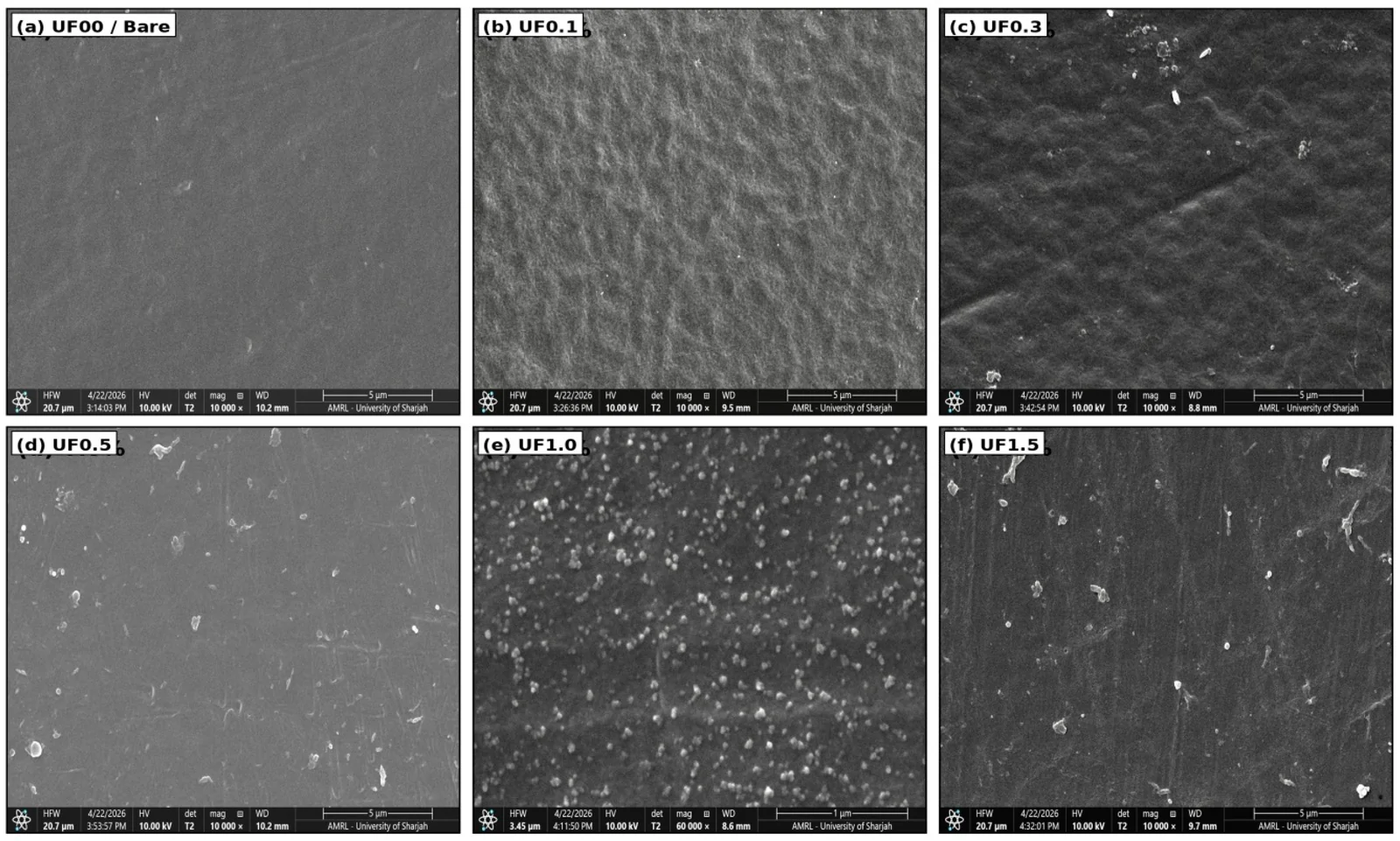
Surface SEM images of pristine and Ag-MBene-modified PES ultrafiltration membranes: (**a**) UF00, (**b**) UF0.1, (**c**) UF0.3, (**d**) UF0.5, (**e**) UF1.0, and (**f**) UF1.5.

The surface morphologies of the prepared membranes were systematically analyzed using FE-SEM studies to evaluate the effect of Ag-MBene incorporation on the structural development of the membranes (see Figure 1 below). The pristine PES membrane had a relatively smooth and dense surface morphology with limited pore distributions. On incorporating Ag-MBene in the membranes, significant changes in their surface morphologies were observed. Increased pore interconnectivity and macrovoid formation indicate accelerated solvent-non-solvent exchange during phase inversion. Among all the prepared membranes, the UF0.5 exhibited the most optimized surface morphology and interconnected pore channels with well-balanced pore distribution. The enhanced pore interconnectivity in the UF0.5 membrane reduced internal hydraulic resistance and facilitated permeation. The morphological evolution suggests a transition from delayed demixing in the pristine membrane to instantaneous demixing in Ag-MBene-modified systems, driven by accelerated solvent-non-solvent exchange during phase inversion. Rasool et al. revealed that the hydrophilic nature of the incorporated Ag-MBene nanoparticles accelerated the exchange rate of NMP solvent with water during the precipitation of membranes, thus promoting porous formation and macrovoid development [18,21].

Similar pore evolution trends have been reported for nanomaterial-modified PES ultrafiltration membranes, where hydrophilic nanostructures accelerate solvent-non-solvent exchange and promote macrovoid development. The pore architecture observed in UF0.5 falls within the morphology range reported for high-performance nanocomposite ultrafiltration membranes.

Similar finger-like macrovoid structures have been widely reported in PES membranes fabricated via NIPS due to rapid solvent-nonsolvent exchange [9,10,22].

At higher nanoparticle loadings, increased dope solution viscosity and nanoparticle agglomeration suppress mass transfer during phase inversion, resulting in reduced pore interconnectivity and partial pore blockage [21].

Previous studies have reported that excessive nanofiller loading can increase casting-solution viscosity, promote nanoparticle agglomeration, and impair nanoparticle dispersion [9,10].

Figure 1 shows the surface morphology of the fabricated membranes. The pristine UF00 membrane exhibited a relatively dense surface, whereas Ag-MBene incorporation modified the pore structure and promoted the formation of more interconnected pores. The UF0.5 membrane exhibited a more uniform porous structure, which contributed to enhanced permeability and fouling resistance.

Meanwhile, increasing nanoparticle loading concentration adversely affected the structural stability of the membranes. As discussed above, enhanced casting solution viscosity owing to increased nanoparticle concentration resulted in partial pore collapse and dense membrane regions limiting water permeation.

#### 3.1.2. Cross-Sectional Morphology

**Figure 2:**
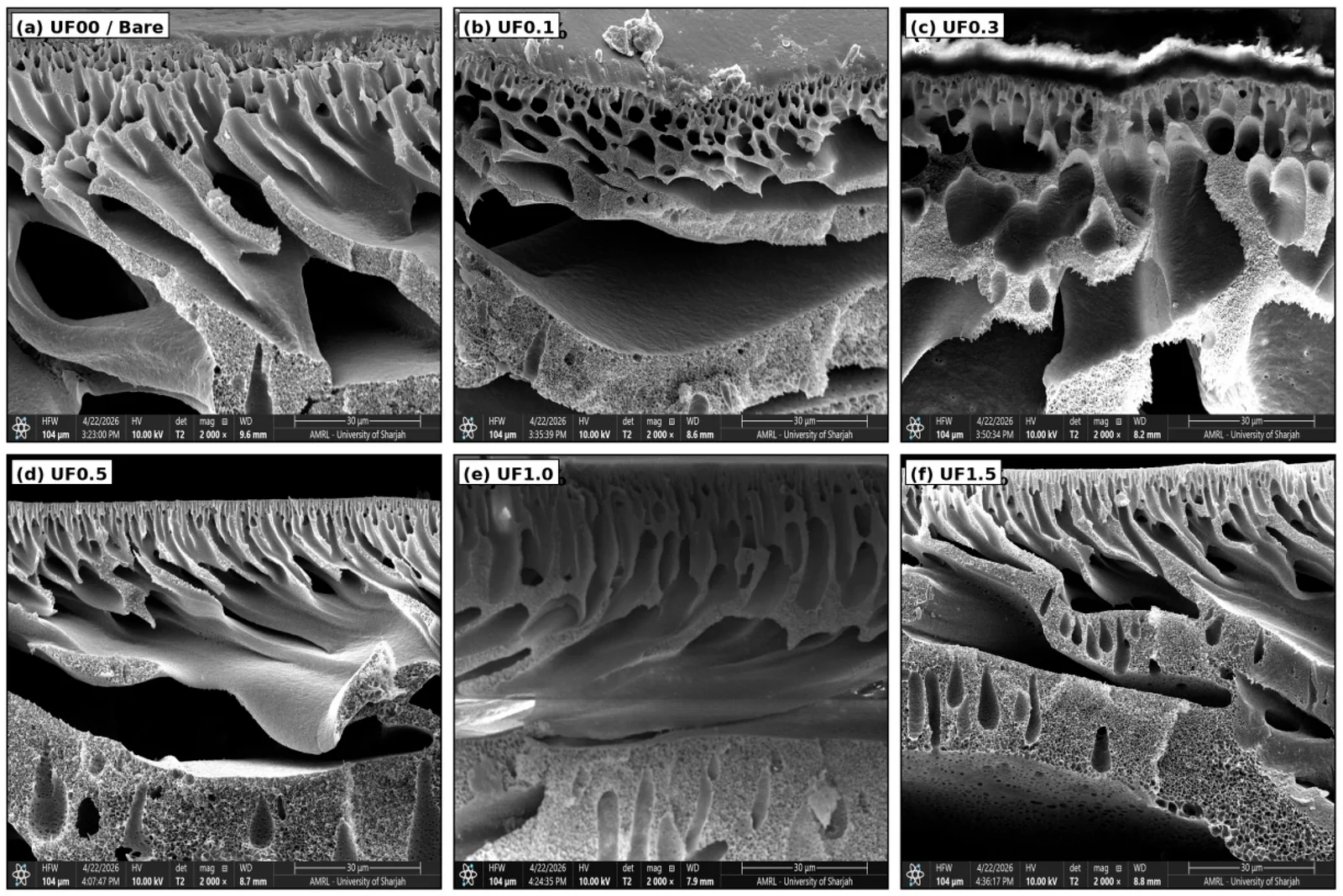
Cross-sectional FE-SEM morphology of Ag-MBene-modified PES membranes.

Figure 2 presents cross-sectional FE-SEM images illustrating the influence of Ag-MBene incorporation on membrane structural development. The pristine membrane exhibited fewer macrovoids and a denser morphology than the modified membranes. As evidenced by SEM results, the unmodified membrane had relatively few macrovoids and was denser than the modified ones.

With respect to the introduction of the nanofillers, it was observed that the modified membranes had relatively distinct finger-like macrovoid structures formed along their entire cross-section. Moreover, the UF0.5 membrane showed optimal cross-sectional morphology in terms of enhanced continuity and distribution of pores and channels formed. This promoted rapid water diffusion into the casting solution, accelerating demixing during phase inversion.

Nevertheless, high amounts of nanoparticles incorporated resulted in an unstable membrane structure and incomplete pore formation. Increased concentration of the nanoparticles enhanced solution viscosity and decreased phase inversion kinetics. From this observation, it can be concluded that optimal membrane performance was achieved only at moderate Ag-MBene loading concentrations, whereas excessive nanoparticle incorporation reduced structural uniformity because of increased casting solution viscosity and nanoparticle agglomeration.

Figure 2 demonstrates that all membranes exhibited asymmetric finger-like structures extending from the bottom layer. Increasing Ag-MBene concentration altered the internal morphology and promoted more pronounced macrovoid formation. The optimized UF0.5 membrane displayed a balanced porous substructure favorable for water transport. These structural changes directly contributed to the enhanced porosity and reduced hydraulic resistance observed later in Section 3.6 and Section 3.8.

### 3.2. AFM Analysis

#### 3.2.1. Surface Roughness Evolution

Atomic force microscopy (AFM) was employed to investigate the surface topography and roughness characteristics of the fabricated Ag-MBene-modified polyethersulfone ultrafiltration membranes. Surface roughness plays an important role in determining membrane wettability, water transport behavior, fouling tendency, and overall filtration performance. The AFM analysis was conducted over a scanning area of 5 μm × 5 μm, and the average surface roughness (Ra) was used as the primary roughness parameter for comparative evaluation.

The pristine UF00 membrane exhibited an average surface roughness (Ra) of 8.27 nm. Following Ag–MBene incorporation, Ra progressively increased to 11.65, 11.98, 14.24, 16.51, and 19.99 nm for UF0.1, UF0.3, UF0.5, UF1.0, and UF1.5, respectively. These results demonstrate that Ag–MBene incorporation progressively increased nanoscale surface heterogeneity. The observed roughness evolution is attributed to changes in membrane surface morphology induced by nanofiller incorporation during phase inversion.

**Figure 3:**
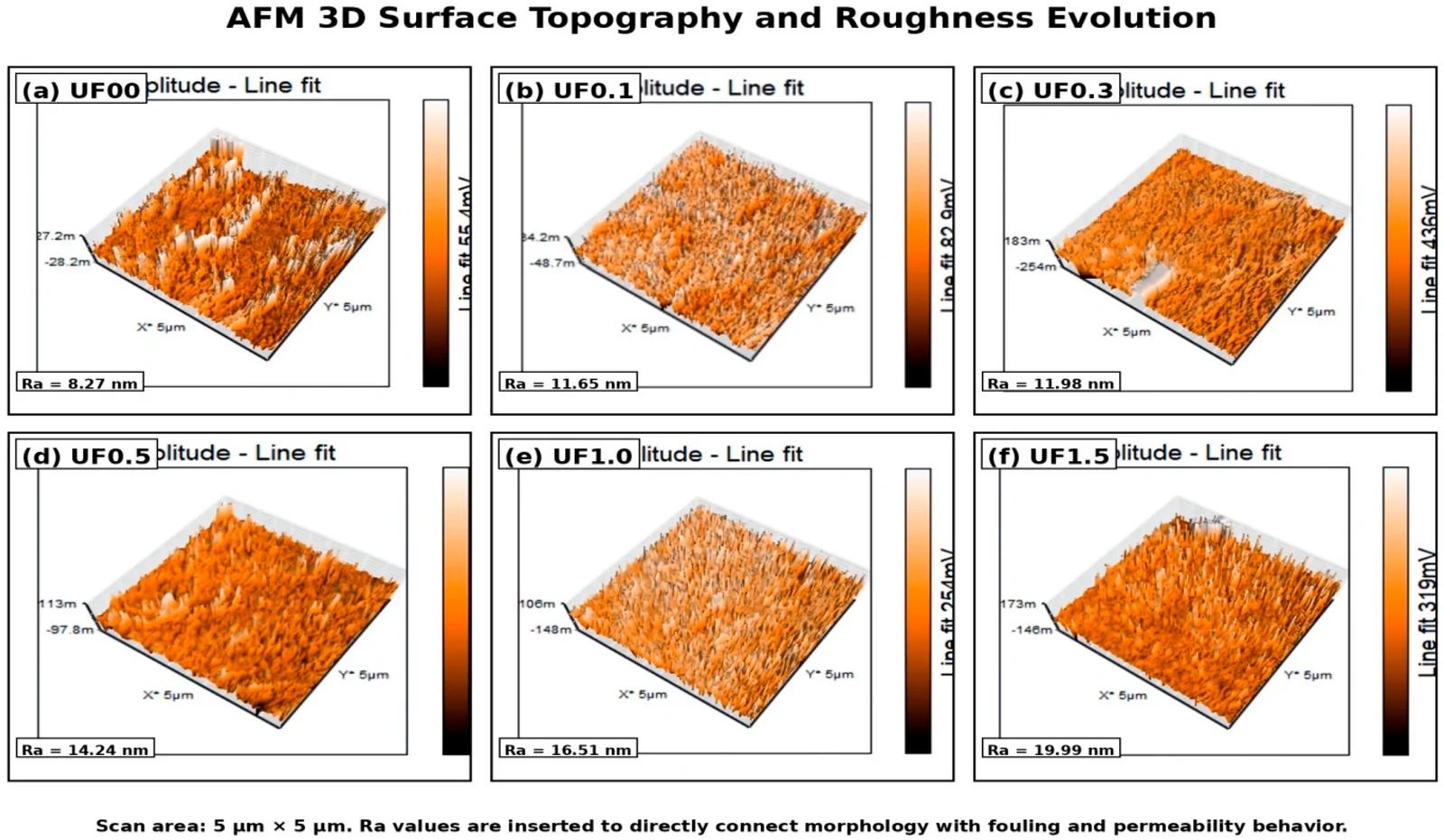
Three-dimensional AFM surface topography of pristine and Ag–MBene-modified PES ultrafiltration membranes: (**a**) UF00, Ra = 8.27 nm; (**b**) UF0.1, Ra = 11.65 nm; (**c**) UF0.3, Ra = 11.98 nm; (**d**) UF0.5, Ra = 14.24 nm; (**e**) UF1.0, Ra = 16.51 nm; and (**f**) UF1.5, Ra = 19.99 nm. AFM measurements were obtained over a scan area of 5 μm × 5 μm.

Figure 3 presents the three-dimensional AFM surface topography of the pristine and Ag–MBene-modified PES membranes. The measured average surface roughness (Ra) increased progressively with Ag–MBene loading, from 8.27 nm for UF00 to 11.65, 11.98, 14.24, 16.51, and 19.99 nm for UF0.1, UF0.3, UF0.5, UF1.0, and UF1.5, respectively. This progressive increase indicates that incorporation of Ag–MBene produced increasing nanoscale surface heterogeneity within the PES membrane.

Importantly, the highest surface roughness was observed for UF1.5 rather than UF0.5. Therefore, the superior overall filtration performance of UF0.5 cannot be attributed to maximum surface roughness. Instead, UF0.5 exhibited an intermediate Ra of 14.24 nm, suggesting that its favorable performance resulted from a balance among surface topography, hydrophilicity, pore architecture, nanoparticle dispersion, and fouling resistance. At higher Ag–MBene loadings, the continued increase in surface roughness did not produce a corresponding improvement in overall membrane performance, indicating that roughness alone is not a sufficient predictor of filtration behavior.

As shown in Table 3, the average surface roughness increased progressively with increasing Ag–MBene loading. Ra increased from 8.27 nm for UF00 to 14.24 nm for UF0.5 and continued to increase to 16.51 and 19.99 nm for UF1.0 and UF1.5, respectively. Thus, UF1.5 exhibited the highest measured surface roughness, whereas UF0.5 exhibited an intermediate roughness value. The superior filtration performance of UF0.5 therefore cannot be attributed to maximum surface roughness alone. Instead, its performance reflects a favorable balance among surface roughness, hydrophilicity, pore architecture, nanoparticle dispersion, and structural stability.

At higher Ag–MBene concentrations (UF1.0 and UF1.5), the average surface roughness continued to increase, reaching 16.51 and 19.99 nm, respectively. However, this continued increase in roughness did not correspond to further improvement in filtration performance. At high nanoparticle loadings, increased surface heterogeneity together with nanoparticle agglomeration and less favorable pore architecture may promote foulant accumulation and reduce effective water transport. Therefore, increasing surface roughness alone should not be interpreted as an indication of improved membrane performance.

The intermediate roughness observed for UF0.5 (14.24 nm) provided a favorable surface topography when considered together with its hydrophilicity, pore structure, porosity, and nanoparticle dispersion. Although UF1.0 and UF1.5 exhibited greater surface roughness, their overall filtration performance declined. This observation further demonstrates that membrane performance depends on the combined effects of surface morphology, wettability, pore architecture, and nanofiller dispersion rather than on maximizing surface roughness.

Comparable changes in surface morphology, wettability, permeability, and antifouling behavior following nanofiller incorporation have been reported for PES nanocomposite ultrafiltration membranes [8,9,10].

The observed roughness trend is consistent with the SEM and EDS analyses, which revealed improved pore development and more uniform nanoparticle distribution at moderate loading levels. The enhanced roughness of UF0.5 contributed to improved wettability and permeability while maintaining structural uniformity.

#### 3.2.2. Roughness-Fouling Relationship

Membrane roughness influences both water transport and foulant deposition behavior. A moderate increase in surface roughness can enhance membrane wettability by increasing the effective surface area available for interaction with water molecules. Consequently, water permeation is facilitated and hydraulic resistance is reduced.

However, excessive roughness may generate surface valleys and localized regions that favor foulant accumulation and irreversible fouling. Therefore, membrane performance depends on achieving an optimal balance between surface roughness and surface uniformity.

The AFM results demonstrate that maximum surface roughness was not associated with maximum membrane performance. Although UF1.5 exhibited the highest Ra value (19.99 nm), UF0.5, with an intermediate Ra of 14.24 nm, exhibited the most favorable overall filtration performance. This indicates that moderate surface heterogeneity can contribute positively to membrane–water interactions when accompanied by suitable hydrophilicity, pore interconnectivity, and uniform nanofiller dispersion. In contrast, greater roughness at higher Ag–MBene loadings may provide additional sites for foulant accumulation and occurs together with less favorable structural changes associated with excessive nanofiller incorporation.

Therefore, surface roughness should not be considered independently when evaluating membrane performance. The superior behavior of UF0.5 is attributed to the combined optimization of surface topography, hydrophilicity, porosity, pore architecture, nanoparticle dispersion, and structural stability rather than to maximum roughness. This interpretation is consistent with the SEM, contact angle, porosity, permeability, and fouling results obtained in the present study.

### 3.3. FTIR Analysis

**Figure 4:**
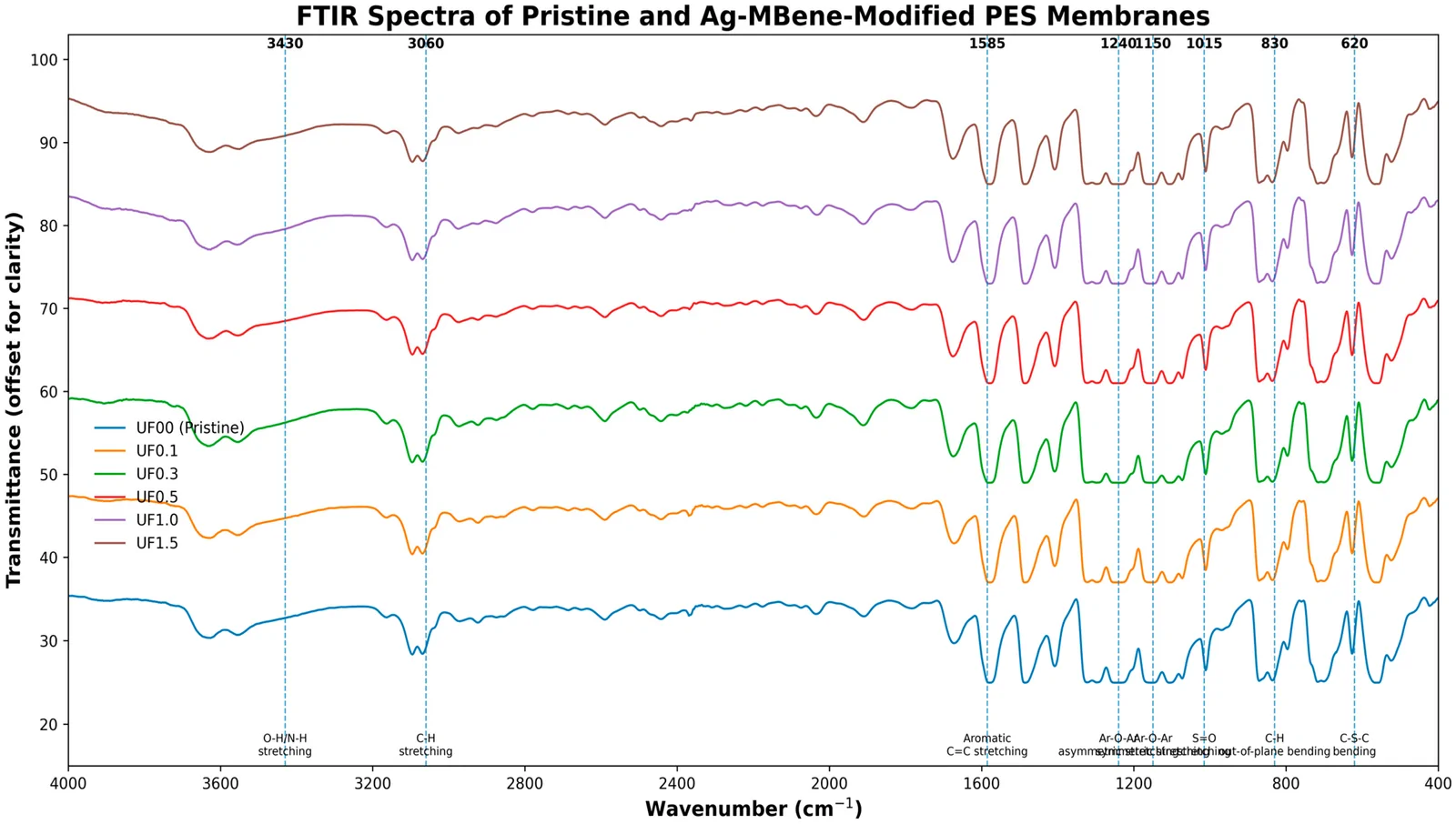
FTIR spectra of pristine PES (UF00) and Ag–MBene-modified PES ultrafiltration membranes (UF0.1–UF1.5). The principal characteristic absorption bands of the PES matrix and the weak low-wavenumber feature observed at approximately 620 cm^−1^ are indicated at their corresponding spectral positions.

The FTIR spectra of the pristine PES membrane (UF00) and Ag–MBene-modified membranes with Ag–MBene loadings of 0.1, 0.3, 0.5, 1.0, and 1.5 wt% are presented in Figure 4. All membranes exhibit the characteristic absorption bands of the PES matrix, confirming that incorporation of Ag–MBene did not alter the fundamental chemical structure of the polymer. Instead, slight variations in peak intensity and transmittance indicate physical interactions between the nanofiller and the polymer matrix.

A broad absorption band centered at approximately 3430 cm^−1^ was assigned to O–H stretching associated with surface hydroxyl groups and adsorbed moisture. The weak band near 3060 cm^−1^ corresponds to aromatic C–H stretching of the PES backbone, while the band at approximately 1585 cm^−1^ is attributed to aromatic C=C skeletal stretching. The characteristic sulfone functionality of PES is represented by bands at approximately 1240 and 1150 cm^−1^, associated with O=S=O stretching vibrations. The band near 1015 cm^−1^ is associated with an S=O-related vibration, whereas the band around 830 cm^−1^ corresponds to aromatic C–H out-of-plane bending. A weak feature was also observed in the low-wavenumber region around 620 cm^−1^. Because this feature is relatively weak and occurs within the fingerprint region, it was not used as definitive evidence for Ag–MBene incorporation.

Compared with the pristine membrane, the Ag–MBene-modified membranes exhibited only minor variations in band intensity, while the principal characteristic absorption bands of PES remained preserved. No major new absorption bands indicative of substantial chemical alteration of the PES backbone were observed. These results suggest that Ag–MBene incorporation predominantly influences the local physicochemical environment of the PES matrix without substantially altering its fundamental chemical structure. The FTIR results should therefore be interpreted together with the SEM and EDS analyses when assessing nanofiller incorporation and distribution.

### 3.4. EDS Analysis

**Figure 5:**
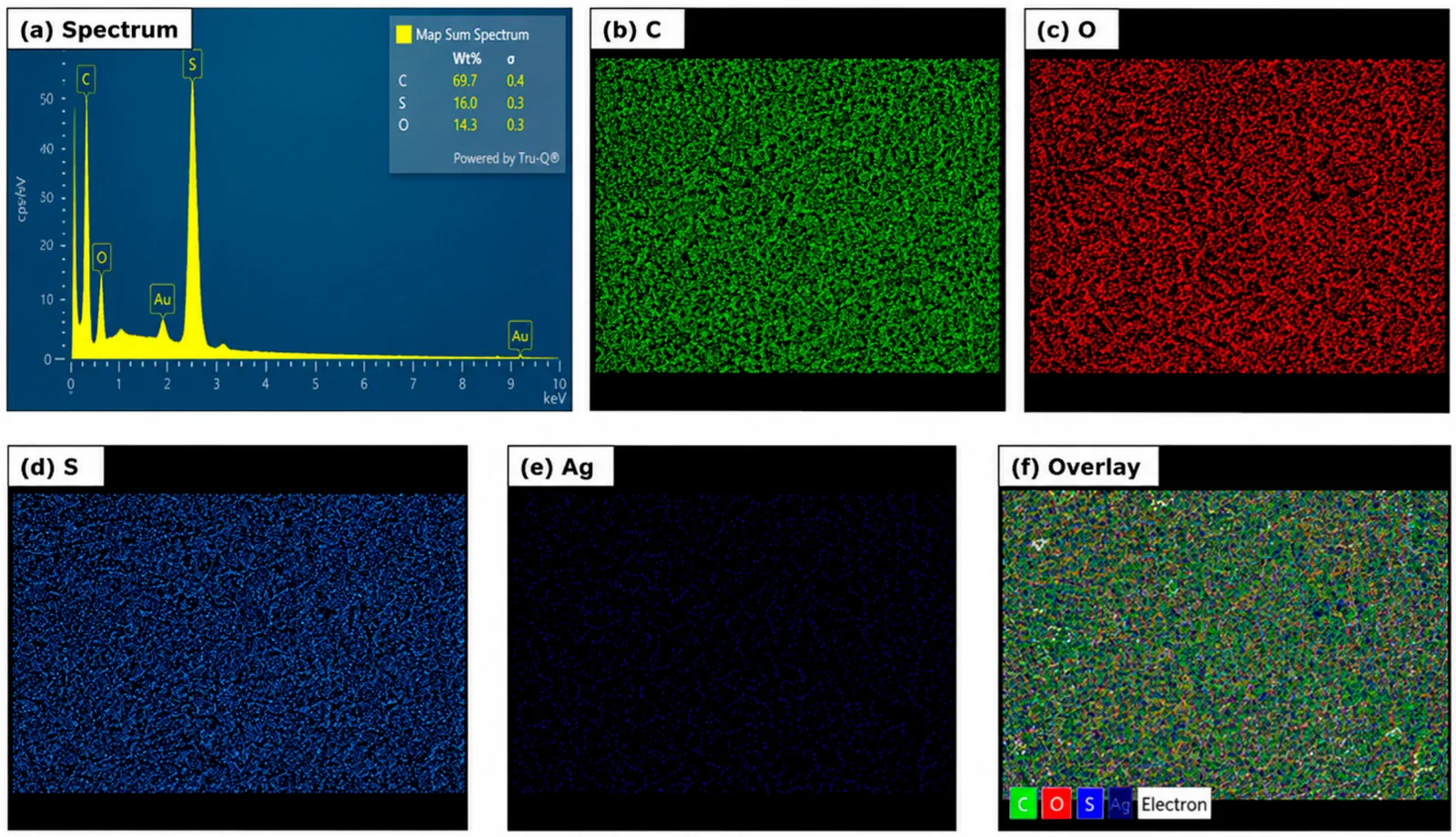
EDS characterization of the Ag–MBene-modified membrane: (**a**) experimental EDS spectrum; (**b**–**e**) elemental maps of C, O, S, and Ag, respectively; and (**f**) elemental overlay. A distinct Ag peak is not clearly resolved in the experimental spectrum under the measurement conditions used; however, the Ag elemental map in panel (**e**) provides complementary evidence of the spatial distribution of Ag within the analyzed region.

Energy-dispersive spectroscopy (EDS) was used to evaluate the elemental composition and spatial distribution of elements within the Ag–MBene-modified membrane, as shown in Figure 5. Carbon, oxygen, and sulfur signals were associated predominantly with the PES/PVP membrane matrix. Because of the relatively low Ag content and the acquisition conditions used, a distinct Ag peak could not be unambiguously resolved in the EDS spectrum. Therefore, the presence and spatial distribution of Ag are evaluated primarily from the corresponding Ag elemental map rather than from assignment of an isolated Ag spectral peak.

EDS analysis confirmed the presence of carbon and oxygen as the dominant elements associated with the PES polymer matrix. The EDS spectrum in Figure 5a is dominated by the major elemental constituents of the membrane. Under the measurement conditions used, the relatively low Ag content does not produce a clearly distinguishable Ag peak. Nevertheless, the Ag elemental map in Figure 5e shows a spatially distributed signal across the analyzed membrane region, providing complementary evidence for the presence and distribution of Ag. The elemental maps further indicate relatively uniform nanofiller distribution within the representative analyzed region.

Furthermore, the elemental distribution maps demonstrated relatively uniform nanoparticle dispersion at lower loadings, whereas slight aggregation became noticeable at higher Ag-MBene concentrations. These observations are consistent with the SEM morphology results and support the improved membrane performance observed for UF0.5.

The elemental map indicated relatively uniform elemental distribution was observed at lower nanoparticle loading, while higher loading resulted in localized agglomeration and clustering within the membrane matrix. It was observed that the most uniform dispersion pattern was achieved in the UF0.5 membrane due to improved compatibility between nanoparticles and the PES matrix material. Improved dispersion behavior was associated with increased membrane hydrophilicity, continuity, and permeability properties [20].

On the other hand, increased nanoparticle concentrations caused aggregation in specific areas, caused by clustering of the nanoparticles within the membrane sample. Nanoparticle aggregation reduced membrane structural uniformity, leading to blockage and decreased membrane permeability.

Carbon and oxygen were the dominant elements originating from the PES matrix and PVP additive. Sulfur peaks corresponded to sulfone groups present in PES. The Ag elemental map provided complementary evidence for the presence and spatial distribution of the Ag-containing nanofiller within the analyzed membrane region. Elemental maps demonstrated relatively uniform Ag distribution in UF0.5, whereas localized clustering became more evident at higher nanoparticle loadings, indicating agglomeration.

Carbon and oxygen were the dominant elements detected and mainly originated from the PES matrix and PVP additive. Sulfur signals corresponded to the sulfone groups characteristic of PES. Although a distinct Ag peak was not clearly resolved in the spectrum, the Ag elemental map showed a distributed Ag signal within the analyzed region, providing complementary evidence for the presence of the Ag-containing nanofiller. The elemental distribution observed in the mapping results indicates nanofiller dispersion within the representative analyzed membrane region. Gold signals observed in the EDS spectrum originate from the conductive gold coating applied before SEM/EDS characterization.

**Figure 6:**
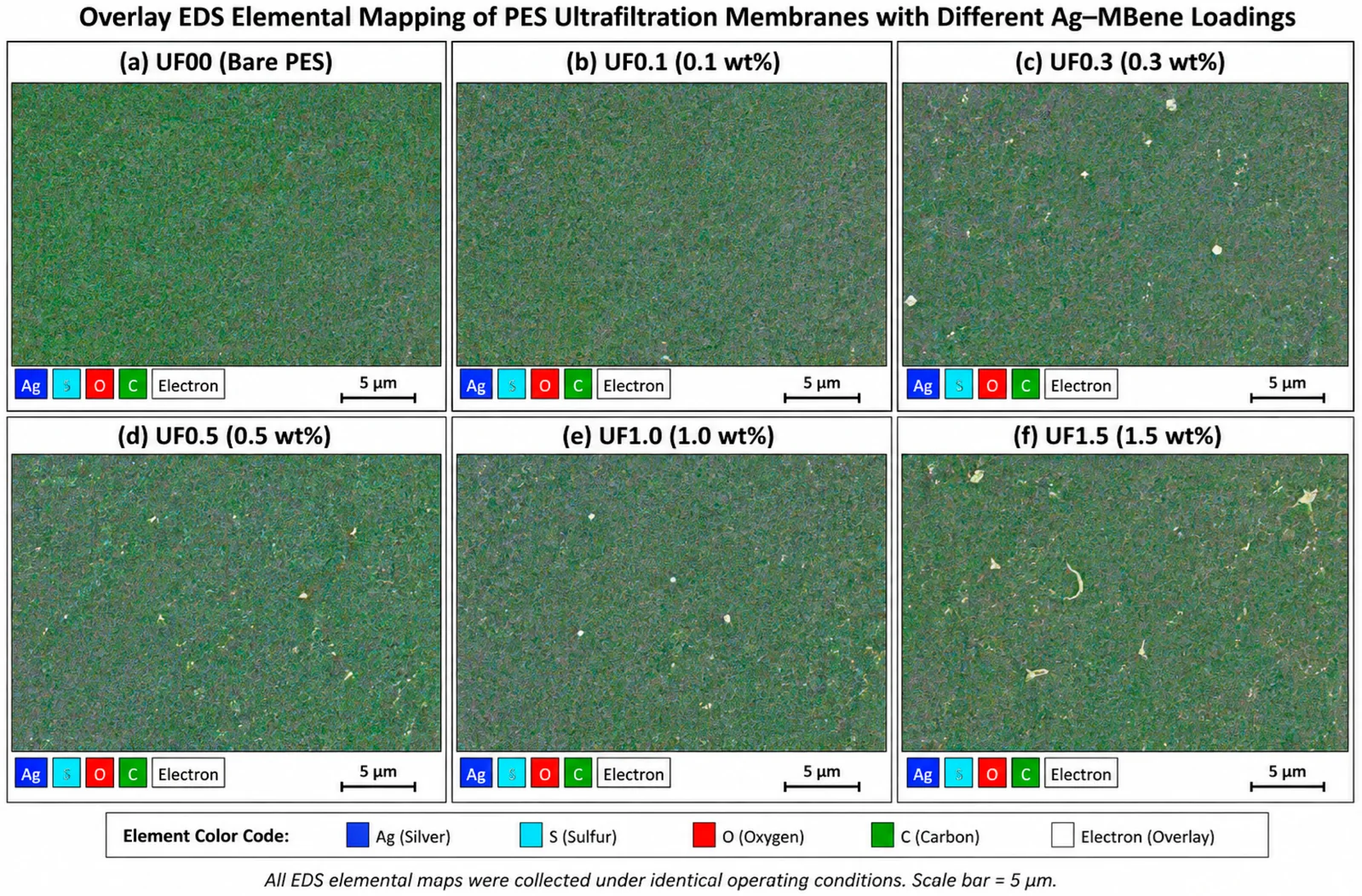
Overlay EDS elemental maps of PES membranes with different Ag-MBene concentrations: (**a**) UF00, (**b**) UF0.1, (**c**) UF0.3, (**d**) UF0.5, (**e**) UF1.0, and (**f**) UF1.5.

As shown in Figure 6, the overlay EDS elemental maps confirm the successful dispersion of Ag-MBene nanosheets within the polyethersulfone matrix. The relatively uniform elemental distribution indicates effective nanoparticle incorporation at moderate loading levels and minimizes the likelihood of severe agglomeration. This homogeneous distribution contributes to the enhanced porosity, permeability, and fouling resistance observed for the optimized UF0.5 membrane. Overall, the dispersion behavior of nanoparticles has a significant effect on membrane performance and structure. The relatively homogeneous nanoparticle distribution is consistent with the enhanced pore development observed in the SEM images and the increased hydrophilicity measured by contact angle analysis, confirming the strong correlation between nanoparticle dispersion and membrane performance.

EDS elemental mapping was performed on representative surface regions selected independently for elemental analysis. Therefore, the mapped regions presented in Figure 6 are not necessarily identical to the SEM fields shown in Figure 1, although the images correspond to the same designated membrane samples. Differences in local morphological appearance between the SEM and EDS mapping images therefore reflect differences in the analyzed surface regions rather than differences in membrane identity.

### 3.5. Contact Angle Analysis

To evaluate the hydrophilicity of the synthesized membranes, water contact angle measurements were used as an indicator. Table 4 indicates that the pristine PES membrane had a fairly high water contact angle as a result of its inherent hydrophobicity. After the incorporation of the Ag-MBene nanoparticles, the water contact angle was noted to decrease gradually with the increase in the concentration of nanoparticles added to the composite material. The modified membranes exhibited lower contact angle values after Ag-MBene incorporation. The greater wettability of modified membranes is associated with the presence of hydrophilic functional groups on the Ag-MBene surface [21]. These functional groups promoted stronger membrane–water interactions with water molecules and water adsorption on the surface of the membrane. Thus, hydrophilicity helped reduce hydrophobic interactions and contamination adsorption on the membrane surface [13].

The progressive reduction in contact angle confirms enhanced membrane hydrophilicity after Ag-MBene incorporation. Increased hydrophilicity facilitates the formation of a hydration layer on the membrane surface, reducing foulant adhesion and contributing to the improved flux recovery ratios observed for modified membranes.

In addition to that, hydrophilic membrane surfaces facilitated rapid spreading and improved permeability and flux recovery efficiency of the membrane during filtration.

**Figure 7:**
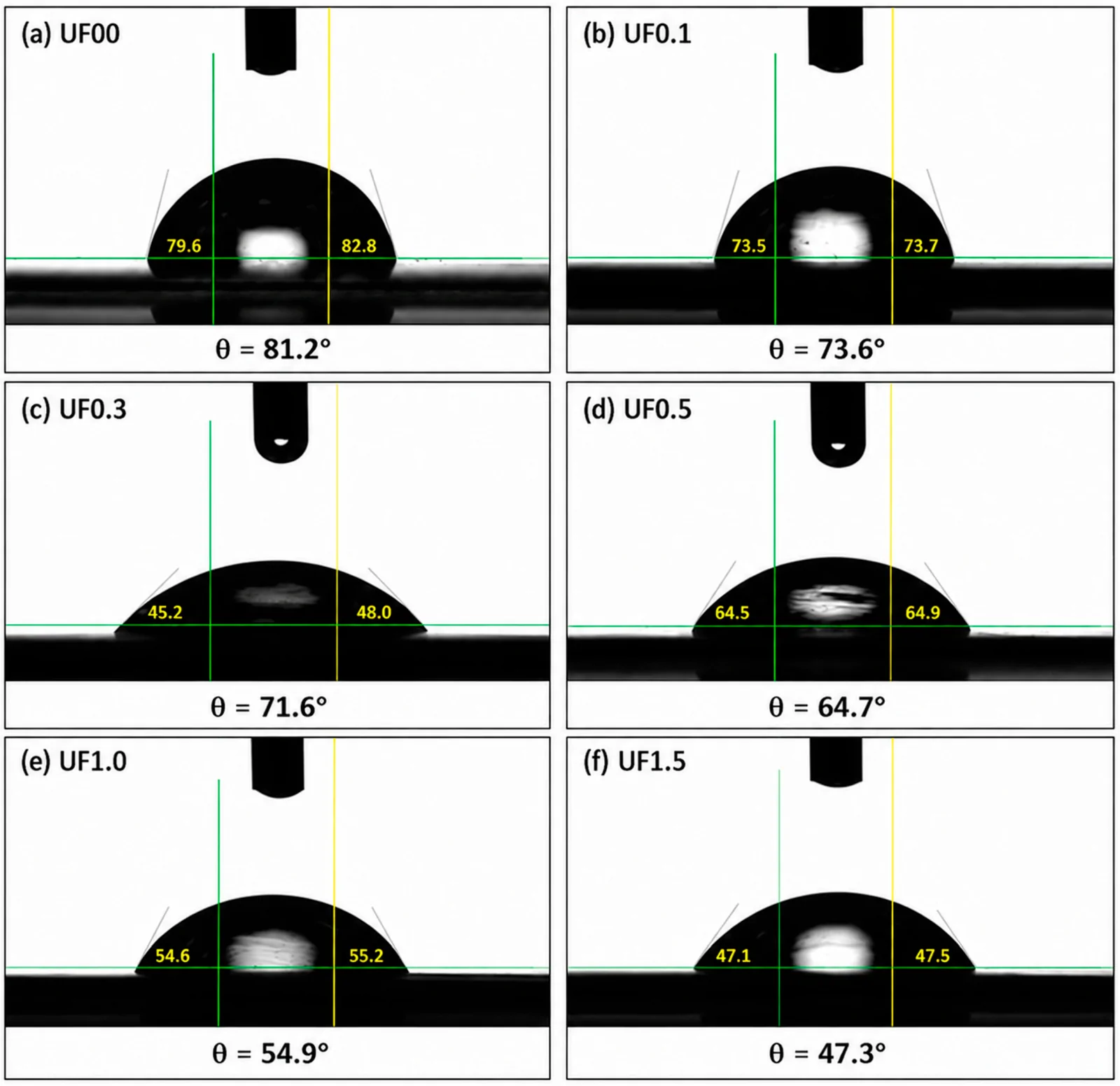
Representative water contact-angle measurements of pristine and Ag–MBene-modified PES ultrafiltration membranes: (**a**) UF00, (**b**) UF0.1, (**c**) UF0.3, (**d**) UF0.5, (**e**) UF1.0, and (**f**) UF1.5.

Figure 7 illustrates the progressive enhancement in membrane wettability with increasing Ag–MBene loading. The water contact angle decreased from 81.2° for UF00 to 73.6°, 71.6°, 64.7°, 54.9°, and 47.3° for UF0.1, UF0.3, UF0.5, UF1.0, and UF1.5, respectively. Thus, UF1.5 exhibited the highest apparent hydrophilicity based on contact-angle measurement. However, the lowest contact angle did not correspond to the highest overall filtration performance. UF0.5 exhibited the most favorable overall performance because its improved wettability was accompanied by optimized pore architecture, porosity, nanofiller dispersion, and structural stability. At higher Ag–MBene loadings, improved hydrophilicity was offset by less favorable structural effects associated with excessive nanofiller incorporation.

Lower water contact angles indicate improved surface wettability and stronger membrane–water interactions, which can facilitate water transport and reduce hydrophobic foulant adhesion. Nevertheless, UF1.5, despite exhibiting the lowest contact angle and therefore the highest apparent hydrophilicity, did not exhibit the best overall filtration performance. This result demonstrates that hydrophilicity alone does not determine membrane performance; pore morphology, pore interconnectivity, surface roughness, nanofiller dispersion, and structural stability must also be considered.

### 3.6. Porosity and Pore Structure Analysis

Membrane porosity, as we can see in Table 5, significantly influences permeability characteristics and hydraulic resistance in filtration processes. The modified membranes had higher porosity than the pristine membrane as a result of the addition of Ag-MBene. The increase in porosity was associated with accelerated solvent-non-solvent exchange induced by the hydrophilic nature of Ag-MBene nanoparticles [21].

The increase in porosity correlated well with the SEM observations of finger-like macrovoid development. UF0.5 had more pore connectivity without compromising the membrane’s structural strength. Increased amounts of nanoparticles also improved membrane porosity. Despite this, at high nanoparticle concentrations, structural instability and partial pore occlusion were observed because of nanoparticle clustering. Therefore, too much porosity improvement did not necessarily translate into improved filtration efficiency. However, the effective transport contribution of porosity is governed not only by total void fraction but also by pore interconnectivity and tortuosity, which collectively evaluate actual hydraulic accessibility. Although increased porosity generally enhances permeability, membrane performance depends on both total void fraction and pore connectivity. The superior performance of UF0.5 indicates that an optimal balance between porosity, pore interconnectivity, and structural stability is more important than maximizing porosity alone. Porosity alone was insufficient to evaluate membrane performance. Instead, membrane permeability depended on the combined effects of pore connectivity, structural continuity, and nanoparticle dispersion. The observed increase in membrane porosity agrees well with the SEM cross-sectional morphology, where accelerated solvent-nonsolvent exchange promoted the formation of interconnected finger-like macrovoid structures that enhanced water permeability. Increased porosity alone does not necessarily guarantee superior membrane performance because excessive pore development may compromise mechanical stability and selectivity.

### 3.7. Mean Pore Size Analysis

The mean pore size of the fabricated membranes, shown in Table 6, was estimated using the Guerout–Elford–Ferry equation. The results demonstrated that moderate Ag-MBene incorporation promoted pore development and improved pore connectivity. The optimized UF0.5 membrane exhibited the largest effective pore size, consistent with SEM observations, enhanced porosity, and increased water permeability.

The increase in pore size up to UF0.5 contributed to reduced hydraulic resistance and enhanced permeability. The enlarged pore network facilitated water transport while maintaining effective pollutant rejection. At higher nanoparticle loadings, partial Ag-MBene agglomeration reduced pore connectivity, resulting in a decline in effective pore size and membrane performance. These findings agree with the SEM and porosity analyses, confirming that pore architecture played a critical role in determining membrane permeability and fouling behavior. The enlarged effective pore radius observed for UF0.5 is consistent with SEM observations and explains the higher pure water permeability measured experimentally. The porosity trend agrees well with the SEM observations, where UF0.5 exhibited the most interconnected pore network, leading to higher permeability and improved flux recovery during filtration.

### 3.8. Flux and Fouling Performance

#### 3.8.1. Comparative Water Flux

The study results revealed considerable improvement in pure water permeability owing to Ag-MBene modification. Nanoparticle incorporation resulted in higher permeance, as shown in Table 7, due to enhanced pore formation and membrane hydrophilicity.

The enhanced permeability behavior resulted from improved hydrophilicity, interconnected pore channels, and reduced hydraulic resistance after Ag-MBene incorporation. Hence, while nanoparticle loading contributed to the formation of hydrophilic surfaces, excess concentrations had a negative effect on permeability performance because of the lack of pore continuity. In addition, viscosity-induced retardation of solvent-non-solvent exchange at higher loadings likely contributes to denser skin layer formation, thereby increasing intrinsic transport resistance despite improved surface hydrophilicity.

Similar nonlinear behavior has been reported in nanocomposite ultrafiltration membranes, where moderate nanoparticle concentrations improve membrane hydrophilicity and pore connectivity, whereas excessive loading increases casting-solution viscosity and promotes nanoparticle agglomeration, leading to pore blockage and reduced permeability. These permeability improvements follow Darcy’s law, whereby reduced hydraulic resistance and increased pore connectivity facilitate greater permeate transport under identical operating pressure conditions. These results demonstrate that permeability is governed by the combined influence of pore morphology, hydrophilicity, and hydraulic resistance rather than by a single membrane property.

#### 3.8.2. Fouling Behavior and Flux Recovery

The fouling behavior of membranes was assessed via flux decline and fouling recovery testing. Table 8 shows that the pristine membrane exhibited poor permeability retention owing to its hydrophobic nature and low fouling resistance capability. There was an increase in fouling resistance after Ag-MBene modification. Flux recovery behavior followed the same trend observed in other performance metrics. The improved fouling reversibility suggests that a greater percentage of foulants were weakly adsorbed on the membrane surface and could be easily removed by flushing. Irreversible fouling was greatly decreased because of the increase in membrane wetting efficiency and the effective formation of the water layer on the membrane surface.

Nevertheless, excessive nanoparticle incorporation increased surface irregularities and promoted foulant accumulation, leading to higher irreversible fouling. This study illustrates the importance of maintaining a balance in improving structural properties and surface characteristics for enhancing membrane antifouling efficiency.

To further quantify fouling behavior, the resistance-in-series model was applied. The total filtration resistance (R_t_) is expressed as:

R_t_ = R_m_ + Rf (9)

where Rt represents total resistance, Rm membrane resistance and Rf fouling resistance according to the resistance-in-series model [10].

The fouling resistance was further divided into reversible and irreversible components. The results indicate that Ag-MBene incorporation significantly reduced irreversible resistance, confirming improved antifouling performance due to enhanced hydrophilicity and reduced foulant adhesion strength. Flux behavior therefore represents the cumulative influence of morphology, pore structure, hydrophilicity, and nanoparticle dispersion established throughout the preceding characterization analyses. Although the present study demonstrates the beneficial influence of Ag-MBene incorporation on membrane hydrophilicity, permeability, and fouling resistance, the release of silver species into the permeate was not evaluated. Since silver-containing nanomaterials may potentially leach during long-term operation, future investigations should quantify Ag release using ICP-OES or ICP-MS under continuous filtration conditions. Such analyses will provide valuable information regarding the long-term environmental safety, membrane stability, and practical applicability of Ag-MBene-modified ultrafiltration membranes.

The present investigation focused primarily on short-term membrane performance and fouling behaviour. Although the optimized UF0.5 membrane demonstrated excellent permeability and flux recovery, long-term cyclic filtration combined with repeated physical and chemical cleaning should be investigated in future studies to assess membrane durability under practical wastewater treatment conditions. Such experiments would provide additional insight into membrane stability during prolonged operation.

### 3.9. COD and TSS Removal Performance

As shown in Table 9, the pollutant rejection performance of the fabricated membranes was investigated through COD and TSS removal analyses during TSE filtration. The modified membranes exhibited enhanced rejection efficiency compared to the pristine membrane.

The improved pollutant rejection efficiency observed for UF0.5 can be attributed to the synergistic effect of enhanced membrane hydrophilicity, optimized pore architecture, and increased foulant resistance. Similar mechanisms have been reported for nanocomposite ultrafiltration membranes treating secondary and tertiary wastewater.

The enhanced rejection performance after Ag-MBene incorporation can be linked to improved membrane hydrophilicity, as shown in Table 10, improved pore distribution, and stronger interactions between the membrane surface and wastewater components.

Furthermore, the modified membranes exhibited higher COD removal efficiency because of improved adsorption and filtration interactions occurring within the membrane structure. Hydrophilic membrane surfaces promoted water transport while restricting larger organic contaminants through the membrane pores. This suggests a transition from predominantly size-exclusion-controlled separation in the pristine membrane to a hybrid adsorption-sieving mechanism in modified membranes under moderate Ag-MBene loading.

Meanwhile, excessive nanoparticle incorporation negatively influenced pollutant rejection stability because of structural irregularities and localized pore blockage. Nanoparticle agglomeration reduced membrane uniformity and created unstable transport pathways that adversely affected separation performance.

Overall, the COD and TSS rejection results indicate that moderate incorporation of Ag-MBene nanoparticles effectively improves wastewater treatment performance through enhancement of membrane structure, hydrophilicity, and filtration stability. Consequently, the improved fouling resistance of UF0.5 cannot be attributed to a single membrane property but rather to the synergistic optimization of morphology, hydrophilicity, roughness, porosity, and pore architecture.

The observed COD reduction is primarily attributed to the combined effects of size exclusion of suspended and colloidal organic matter, adsorption of organic species onto the hydrophilic Ag-MBene/PES membrane surface, and the optimized pore structure developed during phase inversion. The improved pore connectivity and enhanced surface wettability facilitated water transport while simultaneously increasing the rejection of organic contaminants responsible for chemical oxygen demand.

### 3.10. Statistical Analysis

One-way ANOVA was performed to evaluate the statistical significance of performance differences among the fabricated membrane configurations. As summarized in Table 2, statistically significant overall differences were observed for average flux, flux recovery ratio (FRR), COD removal, and TSS rejection (*p* < 0.05). These findings demonstrate that membrane performance varied significantly with Ag–MBene loading. UF0.5 exhibited the most favorable overall experimental performance among the investigated configurations. Nevertheless, because post hoc multiple-comparison testing was not performed, the ANOVA results demonstrate overall differences among the membrane configurations and should not be interpreted as establishing statistical significance for individual pairwise comparisons.

The significant increase in membrane permeability can be associated with enhanced hydrophilicity, improved pore interconnectivity, and lower hydraulic resistance within the membrane structure. Likewise, the statistically significant improvement in fouling resistance and flux recovery behavior demonstrates that membrane surface modification effectively minimized irreversible foulant deposition and enhanced filtration stability. The ANOVA analysis also confirms that moderate nanoparticle incorporation provided the most balanced improvement in overall membrane performance. Future studies may employ Tukey HSD or Duncan multiple comparison tests to identify statistically significant pairwise differences among membrane configurations. Because the primary objective of ANOVA was to verify overall statistical significance among membrane configurations, post hoc comparisons were considered beyond the scope of the present work and are recommended for future investigations.

### 3.11. Integrated Performance Assessment of Membrane Configurations

The consistency of the membrane performance trends was examined by comparing the main experimental indicators, including permeability, fouling resistance, flux recovery, COD removal, TSS rejection, hydrophilicity, porosity, and structural stability. The UF0.5 membrane consistently demonstrated the most balanced performance across these parameters. This confirms that its selection as the optimal membrane configuration was not controlled by a single performance indicator, but was supported by the combined improvement in structural, physicochemical, and filtration characteristics. The UF0.5 membrane exhibited optimized permeability, stable pore morphology, improved fouling reversibility, enhanced recovery behavior, and reliable pollutant rejection efficiency. The superior performance of the UF0.5 membrane confirms that moderate Ag-MBene incorporation provided the most favorable balance between hydrophilicity enhancement, permeability improvement, pore continuity, and structural stability. These findings strongly agree with the experimental observations obtained from SEM, AFM, contact angle, porosity, permeability, fouling, COD, and TSS analyses.

The performance values presented in Table 11 are compiled from previously reported nanocomposite ultrafiltration membrane studies. Direct comparison across different membrane systems should be interpreted with caution due to variations in operating conditions such as transmembrane pressure, feed composition, membrane fabrication parameters, and testing protocols. Therefore, the comparison is intended to provide a qualitative benchmark of relative performance trends rather than strict quantitative equivalence.

PES refers to polyethersulfone, whereas PSf/PSF refers to polysulfone. The abbreviations used for literature systems are retained according to the cited studies, while the membrane fabricated and evaluated in the present work is Ag-MBene/PES.

#### Robustness of the Integrated Performance Assessment

The consistency of the membrane performance trends was examined by comparing the main experimental indicators, including permeability, fouling resistance, flux recovery, COD removal, TSS rejection, hydrophilicity, porosity, and structural stability. The UF0.5 membrane consistently demonstrated the most balanced performance across these parameters. This confirms that its selection as the optimal membrane configuration was not controlled by a single performance indicator, but was supported by the combined improvement in structural, physicochemical, and filtration characteristics.

### 3.12. Proposed Fouling Mechanism

Based on the combined characterization and filtration results, a fouling mechanism can be proposed for the fabricated membranes during TSE filtration. The pristine PES membrane showed severe fouling behavior because of its hydrophobic surface nature and limited pore interconnectivity. Organic pollutants and suspended particles strongly adhered to the membrane surface, leading to rapid permeability loss and irreversible pore blockage. Following Ag-MBene incorporation, membrane hydrophilicity increased considerably, resulting from the hydrophilic functional groups present on the nanoparticle surface. The improved wettability promoted the formation of a hydration layer on the membrane surface, which reduced direct attachment of foulants.

At the same time, enhanced pore interconnectivity and improved macrovoid formation facilitated continuous permeate transport and lowered internal hydraulic resistance. As a result, the modified membranes exhibited improved fouling reversibility because a greater proportion of contaminants remained weakly attached to the membrane surface and could be removed through hydraulic cleaning. Conversely, excessive nanoparticle incorporation caused structural irregularities and localized roughness regions that encouraged foulant accumulation and irreversible fouling formation.

Therefore, membrane fouling behavior is strongly governed by the balance among hydrophilicity enhancement, pore structure optimization, surface roughness evolution, and nanoparticle dispersion stability.

The present study focused on laboratory-scale evaluation of Ag-MBene-modified PES ultrafiltration membranes using real TSE collected from a municipal wastewater treatment plant. Silver dissolution and long-term nanoparticle stability were not quantified in the present work. Future studies should therefore evaluate Ag release under different pH, ionic strength, and operating conditions using ICP-OES or ICP-MS. In addition, controlled synthetic water matrices with different turbidity levels, organic matter concentrations, and ionic strengths should be investigated to further validate membrane robustness under diverse operating conditions. Silver leaching was not evaluated in the present study and should be investigated in future work using ICP-OES or ICP-MS to assess long-term environmental safety. Although the present work demonstrates excellent short-term filtration stability, long-term cyclic filtration and repeated cleaning experiments are recommended to evaluate membrane durability under practical operating conditions.

## 4. Conclusions

Ag-MBene-modified PES ultrafiltration membranes were successfully prepared via the NIPS method and evaluated for TSE filtration. Incorporating Ag-MBene notably enhanced membrane morphology, hydrophilicity, porosity, permeability, fouling resistance, and pollutant rejection. At moderate loading, improved pore interconnectivity and stronger membrane–water interactions were observed, while higher loading led to nanoparticle agglomeration and reduced structural stability.

Among the fabricated membranes, UF0.5 delivered the best overall performance, with a flux of 280 LMH, an FRR of 86.9%, COD removal of 87.6%, and TSS removal of 94.2%. The results indicate a nonlinear dependence of membrane performance on Ag-MBene loading, with an intermediate level providing the optimal balance between permeability, hydrophilicity, and antifouling behavior.

ANOVA results confirmed significant differences between membrane types, and the overall performance assessment identified UF0.5 as the optimal configuration. The integrated structure-property-performance analysis, supported by statistical validation, confirms UF0.5 as the optimal membrane configuration for tertiary wastewater treatment applications.

Although the developed Ag-MBene-modified PES membranes demonstrated promising filtration performance, further work is required to evaluate long-term operational stability, nanoparticle leaching, cleaning efficiency, biofouling resistance, membrane lifespan, life-cycle sustainability, and pilot-scale feasibility. Future studies should also assess membrane performance using feed waters with different turbidity levels, organic matter concentrations, and ionic strengths to validate membrane robustness under broader operating conditions.

## Figures and Tables

**Table 1:** Membrane coding and Ag-MBene loading.

Membrane	PES (wt.%)	PVP (wt.%)	NMP (wt.%)	Ag–MBene (wt.% of Casting Solution)
UF00	16	2	82	0
UF0.1	16	2	81.9	0.1
UF0.3	16	2	81.7	0.3
UF0.5	16	2	81.5	0.5
UF1.0	16	2	81	1.0
UF1.5	16	2	80.5	1.5

**Table 2:** One-way ANOVA results for membrane performance parameters.

Response Parameter	SS	df	MS	F-Value	*p*-Value	Interpretation
Average Flux	12,854.24	5	2570.85	34.82	<0.001	Significant
FRR	1842.50	5	368.50	27.61	<0.001	Significant
COD Removal	960.45	5	192.09	21.33	<0.001	Significant
TSS Rejection	1105.63	5	221.13	18.94	0.002	Significant

**Table 3:** AFM Surface Roughness (Ra) of Ag-MBene-Modified Membranes.

Membrane	Ra (nm)
UF00	8.27
UF0.1	11.65
UF0.3	11.98
UF0.5	14.24
UF1.0	16.51
UF1.5	19.99

**Table 4:** Contact angle measurements.

Membrane	Contact Angle (°)
UF00	81.2
UF0.1	73.6
UF0.3	71.6
UF0.5	64.7
UF1.0	54.9
UF1.5	47.3

**Table 5:** Membrane porosity analysis.

Membrane	Porosity (%)	SD
UF00	62.1	±1.2
UF0.1	67.4	±1.1
UF0.3	72.8	±1.5
UF0.5	78.6	±1.3
UF1.0	70.5	±1.8
UF1.5	64.3	±2.1

**Table 6:** Mean pore size analysis.

Membrane	Mean Pore Size (nm)
UF00	18.4
UF0.1	22.7
UF0.3	27.5
UF0.5	31.8
UF1.0	26.4
UF1.5	21.9

**Table 7:** Average flux performance.

Membrane	Average Flux (LMH)	SD	% Improvement
UF00	142.0	±4.1	-
UF0.1	185.0	±3.7	30.3
UF0.3	242.0	±5.2	70.4
UF0.5	280.0	±4.8	97.2
UF1.0	210.0	±5.9	47.9
UF1.5	166.0	±6.1	16.9

**Table 8:** Flux recovery and fouling resistance analysis.

Membrane	FRR (%)	Reversible Fouling (%)	Irreversible Fouling (%)
UF00	54.2	22.1	45.8
UF0.1	66.4	28.7	33.6
UF0.3	74.8	35.5	25.2
UF0.5	86.9	47.1	13.1
UF1.0	71.2	31.4	28.8
UF1.5	61.7	24.9	38.3

**Table 9:** COD removal efficiency.

Membrane	COD Removal (%)	SD
UF00	61.2	±2.4
UF0.1	70.5	±2.1
UF0.3	79.3	±1.9
UF0.5	87.6	±1.5
UF1.0	75.2	±2.3
UF1.5	66.8	±2.8

**Table 10:** TSS removal efficiency.

Membrane	TSS Removal (%)	SD
UF00	74.6	±2.0
UF0.1	82.1	±1.8
UF0.3	89.5	±1.5
UF0.5	94.2	±1.2
UF1.0	86.4	±1.9
UF1.5	78.7	±2.1

**Table 11:** Comparative benchmarking with reported nanocomposite UF membranes.

Membrane System	Flux Improvement (%)	Fouling Resistance	COD/TSS Removal	Key Limitation	Reference
GO/PSF UF membrane	25–60	Moderate	Moderate	GO agglomeration	[12,17,18]
MXene/PES UF membrane	40–80	Reduced foulant adhesion strength; flux behavior	Good	Nanosheet restacking	[12,17,18]
TiO_2_/PSF UF membrane	20–50	Moderate	Moderate	Limited permeability	[12]
Ag-MBene/PES UF membrane (This work)	97.2	Excellent	Excellent	High loading agglomeration	This work

## Data Availability

Data supporting the findings of this study are available from the corresponding author upon reasonable request.
